# Water: Free Heat and Water as Remedial Agents

**Published:** 1871-09

**Authors:** T. Curtis Smith

**Affiliations:** Middleport, O.


A MONTHLY RECORD OF

*Medicine, Surgery, and the Collateral Sciences.*

EDITED BY J. ADAMS ALLEN, M.D., LL.D.; AND WALTER HAY, M.D.

VOL. XXVIII.—SEPTEMBER, 1871.—NO. 9.

### Original Communications.

ARTICLE I.—*Water: Free Heat and Water as Remedial Agents.* By T. CURTIS SMITH, M.D., Middleport, O.

In the March number of the Northwestern Medical and Surgical Journal, published at St. Paul, Minn., is an article from Dr. Z. C. McElroy of Zanesville, O., on the "Therapeutic Effects of Free Heat and Water—Flooding the System with Hot Water."

The title and idea at first seem novel, but the remedy is of greater importance in a certain class of diseases (?) or pathological conditions, than would, without consideration, be supposed.

When we consider that fully four-fifths of the earth's composition is water, and that it forms by far the largest portion of the vegetable kingdom from which man is supplied with food either *directly* or *indirectly*,* we may have some conception of its importance in forming a large part of the composition of the human organism.

* Indirectly, *i.e.* the animal food we eat is formed in animals by virtue of their appropriation of vegetable food for their sustenance and growth. We are thus supplied with our most highly organized food.

In the physical organism of the human system, water constitutes, of the blood alone, sixteen-twentieths,* and of the whole system nearly nineteen-twentieths. It is not strange, then, that man and animals die much sooner for the want of water alone than for the want of food.

All the secretions are composed very largely of water, as bile, gastric juice, perspiration, etc., and it is the agent which, holding in solution or mixture, by *transudation*, carries nutriment and remedial agents through the system to every part supplied with the circulating fluids, and also carries from every part, however remote or near, the molecular debris of tissue waste, and poisonous material of whatever kind. These are carried out of the system by the excreting organs, water being their principal (in bulk) ingredient.

Much of the so-called mineral water is purer and freer from mineral constituents than our ordinary well or spring water, but a certain class of patients, small drinkers at home, visit these springs and are benefited, simply because at home they did not use enough of this natural element of their bodies, and their physician, giving the subject no thought, did not advise them to do so, while at the springs they drink largely, and the water filters out of their blood and tissues the retained excess of salts and molecular debris of tissue waste. The system thus freed of the baneful effects produced by poisonous elements remaining, especially decaying animal matter, floating in the blood and fluids through the system in microscopic molecular forms, the patient "feels better," and is better.

A notorious instance of this kind is that of the great reformer, Martin Luther.† "The testimony of experience to the use of water as a remedial agent, is shown in the patronage bestowed from the earliest times upon numerous springs whose saline constituents are even less abundant than those of ordinary drinking water. Pfeffers, historically famous for freeing Martin Luther of his demon-haunted hypochondriasis, is still the resort of the invalid. It is situated in a most gloomy hole: and the copious hot stream that boils out of the rock *is almost chemically pure.*‡ So that

* Carpenter's Physiology, p. 185.

† Chambers' Renewal of Life, pp. 565–6; 2nd Am. ed.

‡ The italics are mine.

really the pure nymph of the fountain, innocent of salt, should have the whole credit. The same may be said of the well-known Gastein and Wildbad, the crowded Baden, imperial Plombières, of the French Aix, and our own long-frequented Buxton; for, practically speaking, the influence of the saline particles they contain must be reckoned for nothing. It is certainly nothing as compared with the effects of moderate doses of water in Dr. Böcker's experiments."

As much may be said of many of the "mineral springs" that have become renowned in this country. The water-cure has been looked upon in this country by regular practitioners with discredit and prejudice, for the reason that it has, for the most part, been practiced by a class of perambulating quacks; but we should not forget that everything that is good, either to promote health or relieve the afflicted, may be appropriated by the Regulars —*patents excepted*—without a just charge of irregularity. We are limited to no set of agents.

"To remove from the systemeffete matters which are toxically noxious to healthy life, nothingdoes this so universally as *water*."*

"As physiologists, we cannot be surprised at the benefit derived from the simple expedient of drinking water *beyond the demands* of thirst, in all diseases of *arrested metamorphosis*. Taken several times a day *between meals*, it is a most efficient remedy." †

Let us then give it the sanction of well-grounded authority and not despise it, as Chambers *did not*, because it is very simple.

The classes of patients benefited by this water treatment, are those suffering from deficient secretion of perspiration, urine, bile, etc., in which we find the skin husky and dry, bowels costive, a feeling of languor, slight headache, which at times becomes very severe, etc., all of them giving evidence of arrested metamorphosis and deficient elimination.

"In cases where there is arrest of metamorphosis without organic change in any of the viscera, I find that the weaker the spring the better for the patient." ‡

* Chambers' Renewal of Life, p 48.

† Ibid, p. 566.

‡ Chambers.

In the class of cases above named, there is not sufficient fluid taken and absorbed to allow free, rapid and easy motion of molecular forms, and repair is not up to the physiological standard, simply because the wasted or oxidized molecular forms of tissue are not eliminated to give place to those brought within the grasp of the absorbents, *i. e.*, there is not a sufficient vehicle for the oxidized tissue to be carried out, or the nutrient materials to be carried into the tissues of the body. "Water must be in excess of that required in the structure of the tissues, and without which the said materials cannot be absorbed." *

The want of repair, stands as a cause of debility and pathological conditions of an asthenic character; the retention of oxidized—wasted—molecular forms of animal tissue, and some uneliminated salts, cause dry skin, headache, fevers, etc. Sufficient fluid to eliminate these systemic septics will give room for the absorption of nutrient molecular material, furnished by the digestive process. This accomplished, as it often may be by a few very large draughts of water *between meals*, and the physical processes of repair and waste—nutrition and oxidation—become increased in rapidity to a normal velocity, and health attains.

There is no mistake but that the addition of free heat by "flooding the system with hot water" will rapidly increase the velocity of these processes, especially in the interest of waste and afterwards elimination. And I have no doubt, from actual experience, that Dr. McElroy was correct in using this treatment in the class of cases mentioned by him.

"The conclusive experiments of Dr. Böcker and of Dr. Falck, shows the increase of interstitial metamorphosis by this agent (water) to be in close proportion to the quantity taken, within certain bounds; and all who have heard or read of the agreeable sensations experienced by patients during the water cure, cannot doubt its power of removing morbid accumulations of effete matter in the tissues. In this lies its strength; for the demand for new tissue, as expressed in the sensation of hunger, keeps pace exactly with the extent of the metamorphosis. If this demand is rightly supplied, the result must be a complete renewal of the body." †

* Chambers' Renewal, etc., p. 44.

† Chambers, p. 565.

A few cases to clinically illustrate the advantages of this treatment, and I will close.

Mr. J., æt. 30, laborer; bilious temperament, complexion sallow, average height and build, pulse 70, of moderate fullness and force, habits good, skin dry, slightly above natural heat, urine and perspiration rather scant, costive, has constant feeling of dizziness and weight, with pain in the head, the latter often severe, tongue covered with thick yellowish fur, taste bitterish, has a feeling of hebetude all the time.

℞. Mistura creta ʒj. ter die, as a placebo, and directed that he should drink all the water he could *between meals*, and report again in one week. He did so, and expressed himself as feeling better than for months. The treatment was continued for several weeks with good success, all unpleasant symptoms being dissipated. Since that time (two months) he has been in perfect health, and thinks he is so on account of the water cure.

In this case, molecular waste and elimination were retarded. Water increased these processes by constituting a vehicle to carry out oxidized material floating in the fluids, and by causing increase of all the secretions, thus giving better opportunity for healthy metamorphosis.

Mr. J. N.. merchant, æt. 28; spare build, nervous temperament, energetic; health generally very good, but subject once a fortnight to severe attacks of nervous headache, during which he suffers intensely with pain; skin hot and dry; always has to leave his store when an attack comes on. Has tried many remedies but found none that relieved the attacks, but many that made them worse. Says he can always predict an hour beforehand when the pain will cease, by a feeling in the bowels or bladder, showing that there will soon be an evacuation from one or the other. Ordered him to drink two or three quarts of water as *hot as he could swallow*, as soon as the attack begun. He has done so repeatedly, with immediate relief every time. Thought the remedy such a simple one that at first he was not at all disposed to use it, but uses it every time now.

In my own person I have repeatedly used the remedy as in the above case, for similar reasons and with like results. It always causes free diaphoresis, full and forcible pulse, soon followed by free micturition, and often an evacuation from the bowels, pain very soon relieved.*

In the above cases, the system tolerated the presence of oxidized animal tissue and retained urates to a certain extent, then by a powerful effort to eliminate them, did so, but always caused great suffering before it was accomplished. The hot water, holding free heat, increased molecular metamorphosis and caused rapid elimination by increasing the velocity of molecular forms governed by the two physical processes—nutrition and oxidation—and thus health is almost immediately restored.

One other case: Mary Jane, colored, æt. 16; fleshy, lymphatic temperament, had been ill two days, pulse 100, skin hot and dry, headache very severe, tongue red at edges and tip, furred, bowels costive, catamenia regular; generally has good health. Ordered three quarts of hot water to be drank in as short a space of time as possible, after which she was to take magnesia sulph. ℥j. On my next call, stated that she had sweat profusely after the hot water was taken, and kidneys had acted freely, and felt much better within an hour after my visit; at second visit was almost well.

The principal ideas of this plan, as I conceive it, are, *first*, to increase the elimination of oxidized tissue that is floating through the system in molecular forms, also the various salts, as the urates, phosphates, etc.; *second*, to increase oxidation or waste of tissue by increasing the velocity of the physical processes of organic life; *third*, by virtue of the increased waste, to cause increase of repair, and thus cause a more rapid renewal of the system, by which alone a perfect state of health can attain.

Water alone is a valuable agent in many cases like the first given, but when acute attacks of "so-called disease" come on, in many instances free heat and water will effect relief without further help. It will *not* cure every malady or every case we meet, but is very good when proper discrimination and use is made of it. It is also cheap and always at hand.

"In the employment of hot water as a therapeutic measure, my mental conceptions include, not only the water, as *the vehicle*, but *free heat* as the potential force in the combination. Heat, I

* In my own case, I can stave off an attack of headache, etc., by free use of water—especially of *hot water*.

take to be the motor power or force of the universe, as it certainly is of organic life on our globe. In a certain number of cases, I find that there are not fluids enough in the body to carry on the molecular work of waste and repair, and that, as a result, people are sick." *

* McElroy, Northwestern Journal of Med. and Surg., p. 278.

